# Direct Evidence of Ice Crystallization Inhibition by Dielectric Relaxation of Hydrated Ions

**DOI:** 10.3390/ma14226975

**Published:** 2021-11-18

**Authors:** Xiaoyuan Song, Lisheng Zhong, Jinghui Gao

**Affiliations:** State Key Laboratory of Electrical Insulation and Power Equipment, Xi’an Jiaotong University, Xi’an 710049, China; xiaoyuansongxys@stu.xjtu.edu.cn

**Keywords:** ice crystals, microelectrodes, electric field, dielectric relaxation, hydrated ions

## Abstract

In this paper, the inhibition effect of an alternative current (AC) electric field on ice crystallization in 0.9 wt % NaCl aqueous solution was confirmed thermodynamically with characterization. An innovative experimental and analytical method, combining differential scanning calorimeter (DSC) measurement with an externally applied electric field was created by implanting microelectrodes in a sample crucible. It was found that the ice crystallization, including pure ice and salty ice, was obviously inhibited after field cooling with an external AC electric field in a frequency range of 100 k–10 MHz, and the crystallization ratio was related to frequency. Compared with non-field cooling, the crystallization ratio of ice crystals was reduced to less than 20% when *E* = 57.8 kV/m and *f* = 1 MHz. The dielectric spectrum results show that this inhibition effect of an alternating electric field on ice crystal growth is closely related to the dielectric relaxation process of hydrated ions.

## 1. Introduction

Water–ice phase transition is a common phenomenon in nature, which has never failed to remain an important topic in many research fields. For example, a significant issue is how to avoid the formation of ice crystals in the cryopreservation of biological tissues [1,2,3,4,5,6]. Since biological tissues contain a lot of water, ice crystals formed during the freezing process damage tissues and cells, directly through mechanical crushing or indirectly through solution effects [7,8,9,10]. At present, common methods to eliminate ice crystallization include rapid cooling, adding high concentrations of low-temperature protective agents [7,10,11,12,13,14,15], etc. However, these methods have disadvantages, such as being impossible for large samples or the difficulty in removing the protective agents in subsequent steps [16,17]. Recently, the latest research has revealed that ice crystallization is affected by external fields, such as microwave, ultrasonic, magnetic, and electric fields [18,19,20,21,22,23,24].

In this paper, an innovative experimental and analytical method, combining differential scanning calorimeter (DSC) measurement with an applied electric field was realized by implanting microelectrodes in a sample crucible. The effect of alternative current (AC) electric field on ice crystallization in NaCl aqueous solution was studied with quantitative calculation. The results show that the crystallization ratio of ice crystals is significantly reduced under external AC electric field, varies with frequency, and is related to the relaxation polarization of hydrated ions. This may provide a potential physical approach and theoretical basis for cryopreservation technology.

## 2. Materials and Methods

A 0.9 wt % NaCl aqueous solution, which is also called normal saline, was used as the sample. The corresponding molality was 1.552 × 10^−4^ mol/g, and the osmotic pressure was basically equal to that of mammalian blood plasma. Deionized water was also used as a reference.

**Figure 1:**
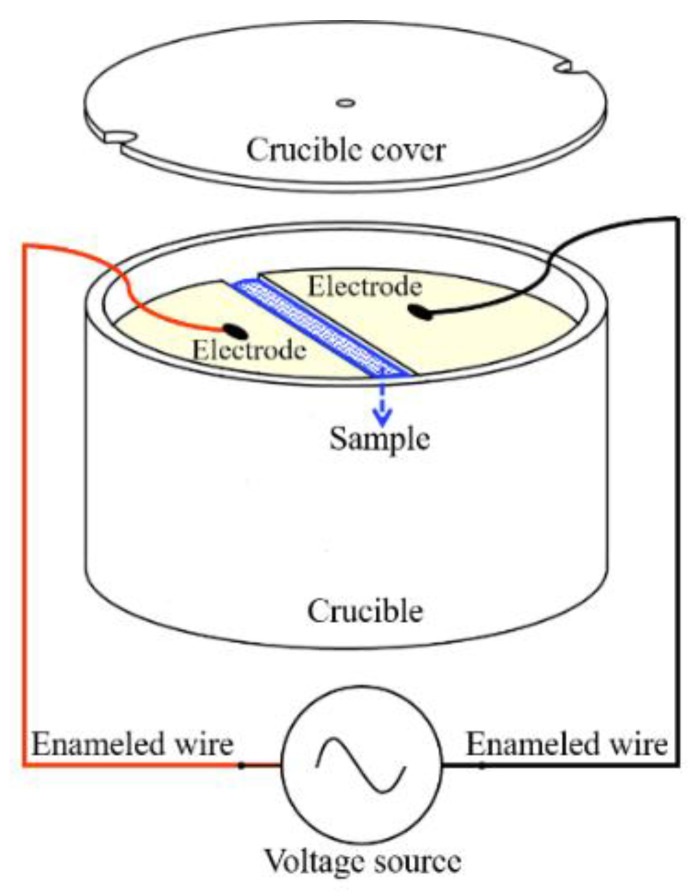
DSC sample crucible with microelectrodes.

In order to ensure that the electric field was applied simultaneously during the DSC (TA Instruments, New Castle, DE, USA) cooling processes, a bespoke crucible was specially designed, as shown in Figure 1. A pair of microelectrodes were placed in a ceramic crucible perpendicularly to the crucible bottom. Two small notches were drilled at the edge of the crucible cover to ensure that the cover could still be placed flat, with enameled wires extending from the crucible. The measured samples were placed in the gap between the microelectrodes. The width of the gap was 350 μm. Microelectrodes were connected to a voltage source providing adjustable sinusoidal AC voltage (*V*_pp_ = 20 V, *E* = 57 kV/m, *f* = 0~10 MHz). The physical and chemical properties of this crucible with microelectrodes remained stable in the whole experimental temperature range and did not affect the performance of other related instruments and the subsequent test results.

**Figure 2:**
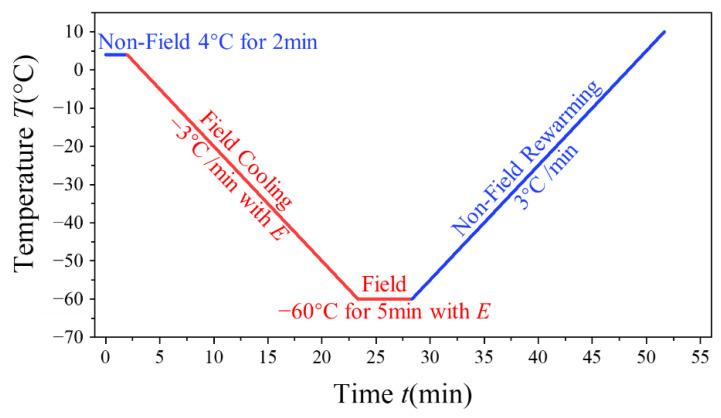
Temperature–time procedure for DSC measurement.

The DSC procedure is illustrated in Figure 2. For the experimental group, i.e., the field cooling group, the procedure was as follows: maintained at 4 °C for 2 min, cooling to −60 °C at 3 °C/min with applied AC electric field *E*, constant temperature at −60 °C for 5 min, turning off the voltage source, and rising to 10 °C at 3 °C/min. The procedure of the control group, i.e., the non-field cooling group, was the same, except that *E* = 0 kV/m during cooling. Deionized water was also tested as a reference in this group. All measurements were repeated three times. In order to compare the results more intuitively and avoid the impact of different sample sizes, 4 μL solution was taken for each test using a micropipette. Rewarming DSC curves were selected for quantitative analysis, since supercooling while freezing brings great interference to the quantitative calculation of cooling DSC curves, and differences in crystallization among samples can be directly reflected through rewarming DSC curves.

The dielectric spectra of 0.9 wt % NaCl solution were also investigated. The complex dielectric constant ε* was measured, and the dielectric loss tangent tan*δ* was calculated [25,26] in the range of 1 Hz–10 MHz from 4 °C to −6 °C.

(1) $$\varepsilon^{*}=\varepsilon^{\prime }-j\varepsilon^{\prime \prime }$$

(2) $$\tan\delta =\varepsilon^{\prime \prime }/\varepsilon^{\prime }$$

*ε*′ is the relative dielectric constant, *ε*″ is the dielectric loss factor, and *δ* is the dielectric loss angle. tan*δ* refers to the energy consumed by the dielectric in converting electric energy into heat energy per unit volume per unit time, and is an attribute of the dielectric itself. In many cases, tan*δ* is much more sensitive to the change of medium characteristics.

A broadband dielectric spectrometer (Novocontrol Technologies, Montabaur, Germany) was used for dielectric measurement. The measurement accuracy of tan*δ* was better than 3 × 10^−5^, and the measurement resolution was less than 10^−5^. For each measured frequency, the output result of the spectrometer was an average value obtained by multiple automatic measurements.

A liquid parallel plate sample cell BDS1308 with two parallel electrodes inside was used, which was sealed to prevent liquid evaporation. The diameter of electrodes was 20 mm. The gap between electrodes was 0.1 mm, adjusted by two silicon dioxide spacers placed in parallel. The stray capacitance of the sample cell was taken into account by open-circuit calibration before measurements. The root-mean-square value of the AC voltage applied to the electrodes was *V*_rms_ = 1 V.

The sample and the sample cell were pre-cooled to 4 °C for 10 min before the start of measurements, to exclude the influence of thermal history on the measurement results. From 4 °C to −6 °C, the dielectric spectrum was measured every 2 °C. The temperature remained constant during the measurement. Each measurement took about 5 min. The temperature control accuracy was ±0.5 °C.

## 3. Results

### 3.1. Variation of DSC Rewarming Curves under AC Electric Field

**Figure 3:**
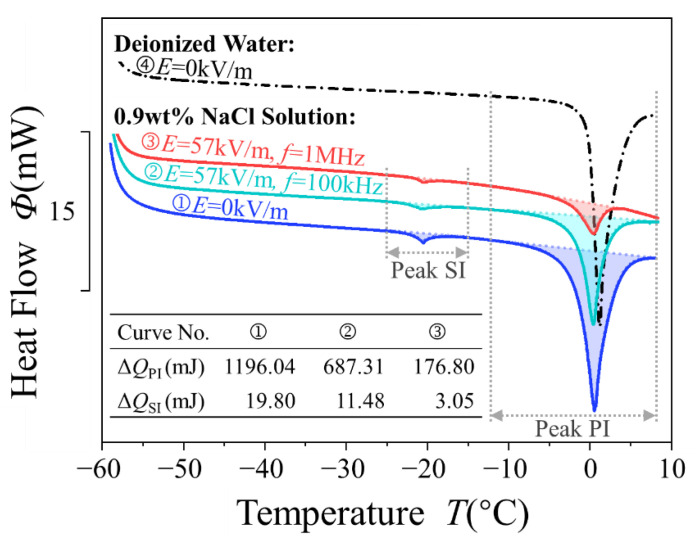
DSC rewarming curves of 0.9 wt % NaCl solution after non-field cooling and field cooling.

Figure 3 shows the rewarming DSC curves of the NaCl solution and deionized water. The sole melting peak of deionized water is caused by pure ice (PI) which is crystals formed by the ordered arrangement of water molecules, and so is the higher temperature melting peak of NaCl solution. The lower temperature melting peak is a eutectic melting peak related to the ions, and is caused by the salty ice (SI), which is a mixture of fine ice crystals and NaCl·2H_2_O [27,28]. During freezing, pure ice crystals first grow around the ice nuclei formed by water clusters. The leftover liquid water molecules and ions near crystal boundaries then tend to form a high-concentration solution, and finally transform into salty ice. Salty ice melts first, and pure ice later, during rewarming.

Compared with non-field cooling, the positions of the salty ice peaks and pure ice peaks remain steady after field cooling, which indicates that the physical properties of pure water ice and salty ice do not change under an electric field, since the melting point is one of the symbolic physical properties of a material.

The area of each peak equals the melting heat ΔQ and can be obtained by integration [29,30]:

(3) $$\Delta Q=\frac{1}{\beta }\int_{T_{1}}^{T_{2}}\Phi dT$$

where Φ is the heat flow, β is the heating rate, and $T_{1}$ and $T_{2}$ are the starting and ending temperature of the peak.

The integration results are given in Figure 3. The ΔQ after field cooling decreased significantly, indicating that the amounts of pure ice and salty ice involved in the melting process were less than after non-field cooling. That is, the external AC electric field hindered the crystallization during the freezing process and provides a promising method to reduce the formation of ice crystals. The possibility remains that the change of ΔQ was due to water transforming into ice with another crystal structure, but previous studies have shown that the electric field mainly affects the water–ice transition through changing the nucleation process, as well as the crystal growth, rather than through variation of ice structure [31,32].

### 3.2. Variation of Crystallization Ratio with Electric Field Frequency

In order to analyze quantitatively, with normalization, the crystallization ratio of pure ice $\eta_{\mathrm{PI}}$ and all ice crystals $\eta_{\mathrm{all}}$ with field cooling and non-field cooling were defined and calculated based on melting heat ΔQ.

(4) $$\eta_{\mathrm{PI}}=\frac{\Delta Q_{\mathrm{PI}}}{\Delta Q_{\mathrm{PI}}^{0}}\;,$$

(5) $$\eta_{\mathrm{all}}=\frac{\Delta Q_{\mathrm{PI}}+\Delta Q_{\mathrm{SI}}}{\Delta Q_{\mathrm{PI}}^{0}+\Delta Q_{\mathrm{SI}}^{0}}\;.$$

$\Delta Q_{\mathrm{PI}}$ and $\Delta Q_{\mathrm{SI}}$ correspond to melting heats after field cooling, while $\Delta Q_{\mathrm{PI}}^{0}$ and $\Delta Q_{\mathrm{SI}}^{0}$ are after non-field cooling. The smaller the $\eta_{\mathrm{PI}}$ and $\eta_{\mathrm{all}}$, the more effective the corresponding applied electric field.

**Figure 4:**
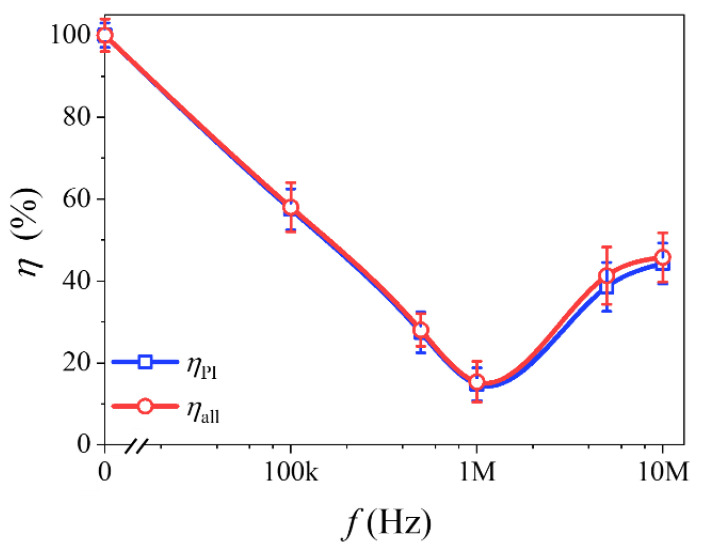
The crystallization ratio of pure ice $\eta_{\mathrm{PI}}$ and all ice crystals $\eta_{\mathrm{all}}$.

The calculation results are shown in Figure 4. It can be seen that the application of an AC electric field decreases $\eta_{\mathrm{PI}}$ and $\eta_{\mathrm{all}}$. The minimum $\eta_{\mathrm{PI}}$ and $\eta_{\mathrm{all}}$ both exist at the optimum frequency *f* = 1 MHz. In addition, the changing trend of $\eta_{\mathrm{PI}}$ and $\eta_{\mathrm{all}}$ is common to both, and the difference between them is unclear. Thus, the effect of an AC electric field on solution freezing is mainly reflected in the inhibition of the growth of pure ice crystals.

### 3.3. Dielectric Spectrum of NaCl Aqueous Solution during Cooling

**Figure 5:**
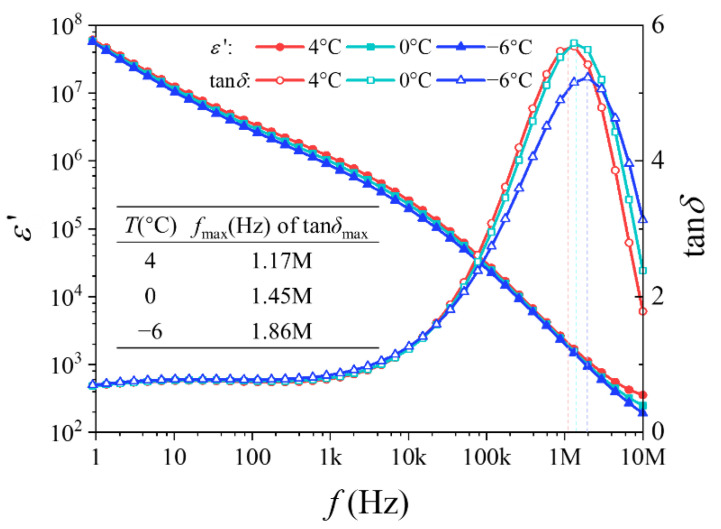
Dielectric spectrum during cooling from 4 °C to −6 °C.

To further explore the mechanism of the strong dependence of $\eta_{\mathrm{PI}}$ and $\eta_{\mathrm{all}}$ on frequency, the dielectric spectra of a 0.9 wt % NaCl aqueous solution during cooling were measured. Since the electric field was applied during freezing for DSC measurement, it is important to pay attention to the dielectric spectrum near the liquid–solid phase transition temperature during cooling, which can be very helpful for discovering the mechanism of the effect of an electric field on crystallization inhibition. Figure 5 shows the dielectric temperature spectra of 0.9 wt % NaCl aqueous solution from 4 °C to −6 °C, close to its liquid–solid transition temperature range of −5.9 to −7.4 °C [33].

The relative dielectric constant $\varepsilon^{\prime }$ decreases very quickly with the increase of frequency, which suggests a relaxation process of hydrated ions. Previous investigations have shown that such a process can be interpreted using the Mangelsdorf and White model [34] from the point view of dielectrophoresis (DEP). The rise of $\varepsilon^{\prime }$ at low frequency can be ascribed to the effect of electrodes [35]. However, the electrode polarization occurs below 1 kHz, which does not interfere with the observation and analysis of the concerned relaxation polarization of hydrated ions.

The dielectric loss tangent tan*δ* has an obvious peak near *f*_max_ = 1 MHz, consistent with the optimum frequency of the DSC study above. This reveals that the effect of an external AC electric field on the freezing process is related to some kind of relaxation polarization. Since the orientation polarization of water molecules and the induced polarization of hydrated ions appear in the range of 0.1–20 GHz [36,37] and 10 kHz–10 MHz [34], respectively, and the relative dielectric constant $\varepsilon^{\prime }$ decreases more rapidly above 10 kHz, the relaxation polarization effect is not caused by the water molecules, but hydrated ions.

Thus, it is inferred that the relaxation polarization of hydrated ions is the main reason for the inhibition of ice crystallization in a solution when freezing under an AC electric field.

## 4. Discussion

To complete the transition from liquid phase to solid phase and integrate into the lattice structure of ice, water molecules need to overcome the constraints of kinetic energy and potential energy generated by thermal motion and overcome the solid–liquid interface energy.

**Figure 6:**
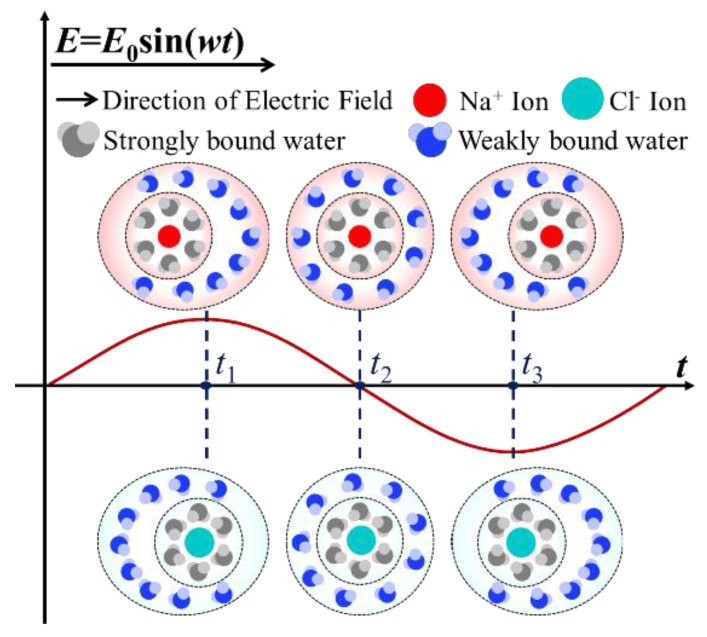
Induced dipoles of hydrated ions under an AC electric field.

In NaCl solution, Na^+^ and Cl^−^ ions are hydrated to form bilayer hydrated ions with opposite charge signs, as shown in Figure 6. The inner layer is strongly bound, and the outer layer is weakly bound. Under an external electric field, the central ion and the strongly bound layer move towards the field direction, and the positive and negative centers of hydrated ions no longer coincide, resulting in induced dipole moments.

Under the application of an AC electric field, the polarization relaxation of hydrated ions destroys the original charge balance, interferes with the original molecular arrangement structure in the solution, and increases the kinetic energy of water molecules because of the friction between the weakly bound water and the water molecules surrounding the hydrated ions; thus, inhibiting the crystal growth of ice.

When the electric field frequency is low enough, the polarization of hydrated ions can keep up with the change of frequency, and the change in direction of the induced dipole is slow. The relatively small disturbance of the solution has a slight influence on the formation and growth process of ice crystals. With an increase of frequency, the polarization gradually lags behind the electric field, resulting in relaxation polarization, the disturbance of the molecular distribution in solution increases, and this has a significant impact on the growth process of ice crystals. With a further increase of frequency, the polarization of hydrated ions cannot keep up with the change of the electric field and can only make small vibrations at the original position; the disturbance is weakened, and the influence on crystallization is also weakened.

Therefore, the influence of hydrated ions on the solution phase transition process under an AC electric field is related to frequency and is the most significant near the range of dielectric relaxation. In addition, this corresponding dielectric relaxation is consistent with the theoretical calculations of the frequency range of induced polarization for hydrated ions in previous works [34,38], which also provide convincing evidence that the ice crystallization inhibition is contributed to by the dielectric relaxation of hydrated ions.

## 5. Conclusions

An innovative crucible with microelectrodes was designed, which enabled a synchronous application of an external electric field during DSC measurements. According to quantitative calculations based on DSC rewarming curves, it was found that ice crystallization in 0.9 wt %NaCl aqueous solution can be effectively inhibited by AC electric field cooling in the range of 100 kHz to 10 MHz. Compared with non-field cooling, the crystallization ratio of pure ice $\eta_{\mathrm{PI}}$ and all ice crystals $\eta_{\mathrm{all}}$ decreases at first, and then increases, with the increase of frequency, when field cooling with an external AC electric field. A window effect appears near *f* = 1 MHz, where $\eta_{\mathrm{PI}}$ and $\eta_{\mathrm{all}}$ are reduced to less than 20%. This frequency is related to the frequency corresponding to the polarization relaxation process of hydrated ions near the liquid–solid transition temperature of the solution. This study may provide a theoretical basis for ice crystal inhibition while freezing and has application potential for the cryopreservation of biomaterials.

## 6. Patents

Two patents resulting from the work are in the application process and are listed as follows:

1. DSC Thermal Analysis Method for Action of Applied Electric Field, Publication No.US-2021-0041381-A1;
2. DSC Electrode System Capable of Applying Electric Field, Publication No.US-2021-0037812-A1.

## Data Availability

The data presented in this study are available on request from the corresponding author. The data are not publicly available due to fund requirements.
